# Medical Society of the County of Erie

**Published:** 1894-09

**Authors:** Franklin C. Gram

**Affiliations:** Secretary


### MEDICAL SOCIETY OF THE COUNTY OF ERIE.

### *Special Meeting.*

Reported by FRANKLIN C. GRAM, M. D., Secretary.

A SPECIAL meeting of the Medical Society of the County of Erie was held at the rooms of the Buffalo Academy of Medicine, on the evening of August 4, 1894, for the purpose of taking action on the death of Dr. Judson B. Andrews, superintendent of the Buffalo State Hospital, and an ex-president of the society. An unusually large number of the profession was in attendance.

After calling the meeting to order, the president, Dr. WM. H. GAIL, of East Aurora, spoke as follows :

It becomes my painful duty to announce the death of one of our most prominent members. Dr. Judson B. Andrews expired at 7.30 o'clock P. M., August 3, 1894, at his home, at the Buffalo State Hospital, after a lingering illness.

The story of his life is too full of important details to be told in this announcement. But few lives, closing at sixty years, have been more completely occupied with honorable, patriotic and useful professional work. His biography, as published in the daily press, leaves little to be added. To us, as a representative body of physicians, it possesses an unusual interest. As our president, in 1886, he received the highest compliment we can bestow, and his many years of service as superintendent of the State Hospital have made him familiar to us all. His fame is not confined to this city, but has extended to distant lands.

He was a striking example of physical and intellectual manhood ; but, more than this, he illustrated in his life the highest ideal of the citizen and the moralist. Among the names of those who have had membership in this society, many of them of world-wide renown, his must ever occupy a most honorable place.

We part from him with deep regret. We shall miss him professionally and socially. But, as the best bequest he could give us, he has trained up men to be his successors in the important specialty to which he consecrated his life. Our hearts throb heavily at the portals of his grave.

It remains for the society to take such action as may seem appropriate, and the chair will entertain suggestions or motions from the members, inviting them also, without special designation, to individually express their sentiments upon this sad occasion.

A committee, consisting of Dr. John Cronyn, Dr. Floyd S. Crego, Dr. W. W. Potter, Dr. F. W. Bartlett and Dr. Roswell Park, was appointed to draft a suitable memorial. The committee reported as follows:

### MEMORIAL.

### Judson Boardman Andrews, A. M., M. D.

Born April 25, 1834.

Died August 3, 1894.

Dr. Andrews passed his early life in New England, and graduated from Yale College in 1855. He then studied medicine, but before receiving his medical degree the civil war began, and he joined the Union Army, serving first as captain in the 77th New York Volunteers. Resigning this office, by reason of impaired health, in 1862, he completed his medical studies and graduated at the Yale Medical School in 1863. Soon afterward he was appointed assistant surgeon in the 2d Connecticut Heavy Artillery, in which he served until the close of the war. In 1867, he was appointed third assistant physician at the State Hospital in Utica, and four years later became first assistant physician to Dr. John P. Gray, serving in this capacity until the Buffalo State Hospital for the Insane was established. He came to Buffalo in 1880, and assumed the superintendency of the latter institution, serving as such until his death. In 1886, he was elected president of this society, serving with distinction. He was president of the New York State Medical Association in 1891, and of the Medico-Psychological Association in 1892.

Dr. Andrews was a man of sound learning, amiable character, sterling integrity and positive convictions. He was fitted by Nature for the work to which he gave the best years of his life, and he easily took rank among the foremost alienists of the world.

With great kindness of heart and manner, he yet governed the unfortunates under his special care with firmness, and often conducted doubtful cases to successful cure.

He was a natural organizer, and so became instrumental in inaugurating methods that resulted in great benefit to the insane and which led to the present improved system.

The medical profession has suffered a blow in his death not easily recovered from, while community is shocked thereby to an unusual degree.

This society desires to express its great appreciation of Dr. Andrews' services to humanity, and its deep sympathy with his surviving widow and daughter in their great affliction.

It, therefore, directs that a copy of this memorial signed by the president and attested by the secretary, be sent to Mrs. J. B. Andrews and published in the medical and newspaper press.

Signed, JOHN CRONYN,
F. W. BARTLETT,
WILLIAM WARREN POTTER,
ROSWELL PARK,
FLOYD S. CREGO,
*Committee.*

Dr. F. S. Crego, in speaking on the adoption of the memorial, alluded to his personal associations with Dr. Andrews. He referred with pleasure to the friendly instructions and advice which the deceased gave him, and how he was the friend of the young man. His kindness toward the patients entrusted to his care at the hospital was proverbial. There was hardly a patient in the large institution whose case he did not understand thoroughly, and who did not love him in return.

Dr. Lucien Howe said that it is customary to take cognizance of a break in the ranks of the profession, but the present was one of unusual importance, having lived and worked among us in so prominent a manner for so long a time. From whatever side we view his character, whether as a citizen, soldier, physician, or as an executive officer, it bears favorable criticism. He alluded to the time when he first met the deceased, and to his future associations with him. Dr. Andrews was quiet and unassuming in his executive position, but when the occasion required he would rouse up and be equal to any emergency.

Dr. Roswell Park spoke of his associations with Dr. Andrews, socially, professionally and in the faculty of the Buffalo Medical College. He alluded to his scientific contributions, and believed him a model for others ; also to the many unjust assaults and criticisms made upon him in his public position. It was unfortunate that such criticisms were ever made, although it is something that public officials are necessarily subjected to.

Dr. W. W. Potter said that as president of this society Dr. Andrews was a model presiding officer, as a specialist he was recognized as an authority, as a superintendent of an insane hospital he was without a superior. His home life, too, was a beautiful one. Take it all in all, we shall rarely be called upon to take cognizance, as a society, of so sad an event as the present.

Dr. Benjamin G. Long said he was a student at the Buffalo University when Dr. Andrews delivered his first course of lectures there, and he referred with pleasure to the deep interest which he took in the young men. He also had occasion for a long time to visit the hospital several times weekly, and he never heard any of the patients speak other than well of him.

Dr. Bartlett briefly alluded to his associations with the deceased; said it was largely due to the late Dr. White that we have the hospital here, and also to him that Dr. Andrews was selected as its superintendent.

Dr. Cronyn said he knew him since he came to Buffalo, and intimately for the past three years, owing to his position as a member of the board of management. His executive work was referred to, and it was his great work which sent Dr. Andrews to an untimely grave.

Dr. Crego offered to present a large photograph of Dr. Andrews to the society. This was accepted, and the memorial as presented was adopted.

Dr. Park said that Dr. Crego had a memorial, which, coming from a former assistant, would be appropriate to incorporate in the minutes. At his request it was read, and on motion spread upon the minutes. It is as follows:

In the death of Dr. Andrews we have sustained a great loss. His kindly disposition and admirable personal qualities have endeared him to all. His learning and devotion to his profession placed him at the head of the specialists in mental diseases at home; and abroad he was accorded a high place in this branch of medicine. We are all under obligation to him for his many contributions to scientific medicine, which we frequently had the opportunity of hearing from his own lips, and the profession at large has recognized their true worth. He was often quoted as an authority on the action of drugs and insanity.

Not less brilliant than his medical career was his army record. When a boy, he was chosen as captain of a volunteer company, went to the front and served for two years, until, on account of failing health, he was obliged to return; but, imbued with patriotism and love for his country, which his ancestors had helped to found, he again went to the front, this time as an assistant surgeon, and remained until the cause of his country had triumphed. The institution of which he was the head was recognized as among the foremost in the country, and his devotion to suffering humanity won for him the love of the patients under his care. The trying labors of his position undoubtedly contributed to his illness, and it can be truthfully said of him that he sacrificed his life to them. He has gone to realize the hope, which as a true Christian he has sustained, of a blissful immortality, mourned by the profession, by the community at large, and by his family, for a more ideal and loving husband and father could not be.

The society then adjourned.

